# Functional connectivity subtypes of MDD and their associations with gene expression profile, neurotransmitter, and cognition

**DOI:** 10.1192/j.eurpsy.2024.746

**Published:** 2024-08-27

**Authors:** Q. Li, Y. Wang, F. Long, Y. Chen, Y. Wang, Q. Gong, F. Li

**Affiliations:** ^1^Department of Radiology and Huaxi MR Research Center (HMRRC), Functional and Molecular lmaging Key Laboratory of Sichuan Province, West China Hospital, Sichuan University, Chengdu, China

## Abstract

**Introduction:**

There’s large heterogeneity present in major depressive disorder (MDD) and controversial evidence on alterations of brain functional connectivity (FC), making it hard to elucidate the neurobiological basis of MDD. Subtyping is one promising solution to characterize this heterogeneity.

**Objectives:**

To identify neurophysiological subtypes of MDD based on FC derived from resting-state functional magnetic resonance imaging using large multisite data and investigate the differences in genetic mechanisms and neurotransmitter basis of FC alterations, and the differences of FC-related cognition between each subtype.

**Methods:**

Consensus clustering of FC patterns was applied to a population of 829 MDD patients from REST-Meta-MDD database after data cleaning and image quality control. Gene transcriptomic data derived from Allen Human Brain Atlas and neurotransmitter receptor/transporter density data acquired by using neuromap toolbox were used to characterize the molecular mechanism underlying each FC-based subtype by identifying the gene set and neurotransmitters/transporters showing high spatial similarity with the profiles of FC alterations between each subtype and 770 healthy controls. The FC-related cognition in each subtype was also selected by lasso regression.

**Results:**

Two stable neurophysiological MDD subtypes were found and labeled as hypoconnectivity (n=527) and hyperconnectivity (n=299) characterized by the FC differences in each subtype relative to controls, respectively. The two subtypes did not differ in age, sex, and scores of Hamilton Depression/Anxiety Scale.

The genes related to FC alterations were enriched in ion transmembrane transport, synaptic transmission/organization, axon development, and regulation of neurotransmitter level for both subtypes, but specifically enriched in glial cell differentiation for hypoconnectivity subtype, while enriched in regulation of presynaptic membrane and regulation of neuron differentiation for hyperconnectivity subtype.

FC alterations were associated with the density of 5-HT2a receptor in both subtypes. For hyperconnectivity subtype, FC alterations were also correlated with the density of norepinephrine transporter, glutamate receptor, GABA receptor, 5-HT1b receptor, and cannabinoid receptor.

Both subtypes showed correlations between FC and categorization, motor inhibition, and localization. The FC in hypoconnectivity subtype correlated with response inhibition, selective attention, face recognition, sleep, empathy, expertise, uncertainty, and anticipation, while that was related to inference, speech perception, and reward anticipation in hyperconnectivity subtype.

**Conclusions:**

Our findings suggested the presence of two neuroimaging subtypes of MDD characterized by hypo or hyper-connectivity. The two subtypes had both shared and distinct genetic mechanisms, neurotransmitter receptor/transporter profiles, and cognition types.

**Disclosure of Interest:**

None Declared

